# Introduction of pediatric laparoscopic inguinal hernia repair in Guatemala

**DOI:** 10.1186/s12893-023-02262-6

**Published:** 2023-11-27

**Authors:** Juan P. Cóbar, Peter F. Nichol

**Affiliations:** 1 Department of Medical Research, Universidad Francisco Marroquín, 6ta calle final zona 10, Guatemala City, 01010 Guatemala; 2 Division of Pediatric Surgery, Department of Surgery, University of Wisconsin Hospitals and Clinics, 600 Highland Ave, Madison, WI 53792 USA

**Keywords:** Pediatric inguinal hernia, Laparoscopic inguinal hernia repair, Low-middle-income country, Patkowski-Takehara technique, Inguinal hernia repair, Pediatric surgery

## Abstract

**Purpose:**

Introducing new surgical techniques in a developing country can be challenging. Inguinal hernias in children are a common surgical problem, and open repair is the standard surgical approach. Laparoscopic repair has gained popularity in developed countries because of similar results. This study aimed to determine the outcomes following the introduction of laparoscopic repairs in Guatemala.

**Methods:**

This retrospective analysis of prospectively collected data from all patients under 18 years who underwent laparoscopic repair at Corpus Christi Hospital in Patzun, Guatemala, from September 5th to September 8th, 2022.

**Results:**

A total of 14 patients were included in the study. A board-certified pediatric surgeon and a Guatemalan physician performed all cases. The mean patient age was 7.6 years; 7 boys and 7 girls. All patients were interviewed at 7 days, 30 days, and 6 months. There were no postoperative infections, pain requiring re-evaluation, gonadal atrophy, or hernia recurrence.

**Conclusion:**

Under controlled circumstances with limited but proper equipment and disposables, laparoscopic inguinal hernia repairs can be introduced and performed in a developing country with a risk complication profile comparable to that in developed countries. This study provides promising evidence of laparoscopic repair feasibility and safety where surgical resources are limited.

**Supplementary Information:**

The online version contains supplementary material available at 10.1186/s12893-023-02262-6.

## Introduction

Inguinal hernias are one of the most common surgical conditions in the pediatric population, with a 0.8-5% incidence in infants and young children [1, 2]. Inguinal hernias are frequently asymptomatic but can cause abdominal pain, discomfort, and complications such as bowel obstruction and strangulation. Surgery is the primary treatment for inguinal hernias, and laparoscopic repair has become increasingly popular among pediatric surgeons in developed countries [1–4].

Open surgical repair with high ligation of the sac had been the standard of care in the United States but has been displaced over the last 2–3 decades by laparoscopic inguinal hernia repair in adults and, more recently, in children [1–3]. Unlike in adults, high ligation of indirect inguinal hernias, even in patients under 18 [5], remains the preferred method of repair in pediatric patients instead of mesh placement. A significant advantage of laparoscopic repair in adults and children is that it allows for examining the contralateral inguinal ring, which may harbor an occult patent processus vaginalis or even a small hernia. Another advantage of the laparoscopic approach in children is that there is no dissection or manipulation of the components of the inguinal canal [3, 6–8].

Additionally, in children, the laparoscopic approach is associated with reduced postoperative pain, shorter hospital stays, quicker recovery time, and a prompter return to school [9]. While there are numerous clinical advantages to laparoscopic inguinal hernia repair, adopting this technique has been slower in developing low- and middle-income countries (LMICs) due to a need for more resources and surgical experience [10] This case series examines the introduction and the outcomes of pediatric laparoscopic inguinal hernia repairs in an LMIC, specifically in Guatemala.

## Methods

### Study design

This was a retrospectively designed, prospective study. We obtained approval for this study from the Ethics Board and performed it under the guise of the International Esperanza Project. The study was determined to be of minimal risk. Data on patient demographics, surgical details, and outcomes were collected from medical records. Inclusion criteria were pediatric patients who underwent laparoscopic inguinal hernia repair under 18 years of age and were diagnosed with inguinal hernia on physical examination. We performed pediatric laparoscopic inguinal hernia repairs using a modified extraperitoneal percutaneous closure based on the Patkowski-Takehara technique at Corpus Christi Hospital in Patzun, Guatemala, over one week, September 4th -7th, 2022. Patzun is a small community located in the Chimaltenango Province of Guatemala. The populace of this region is highly underserved to the point that our group (International Esperanza Project) was the first to conduct pediatric surgery in it.

### Surgical procedure

**Fig. 1:**
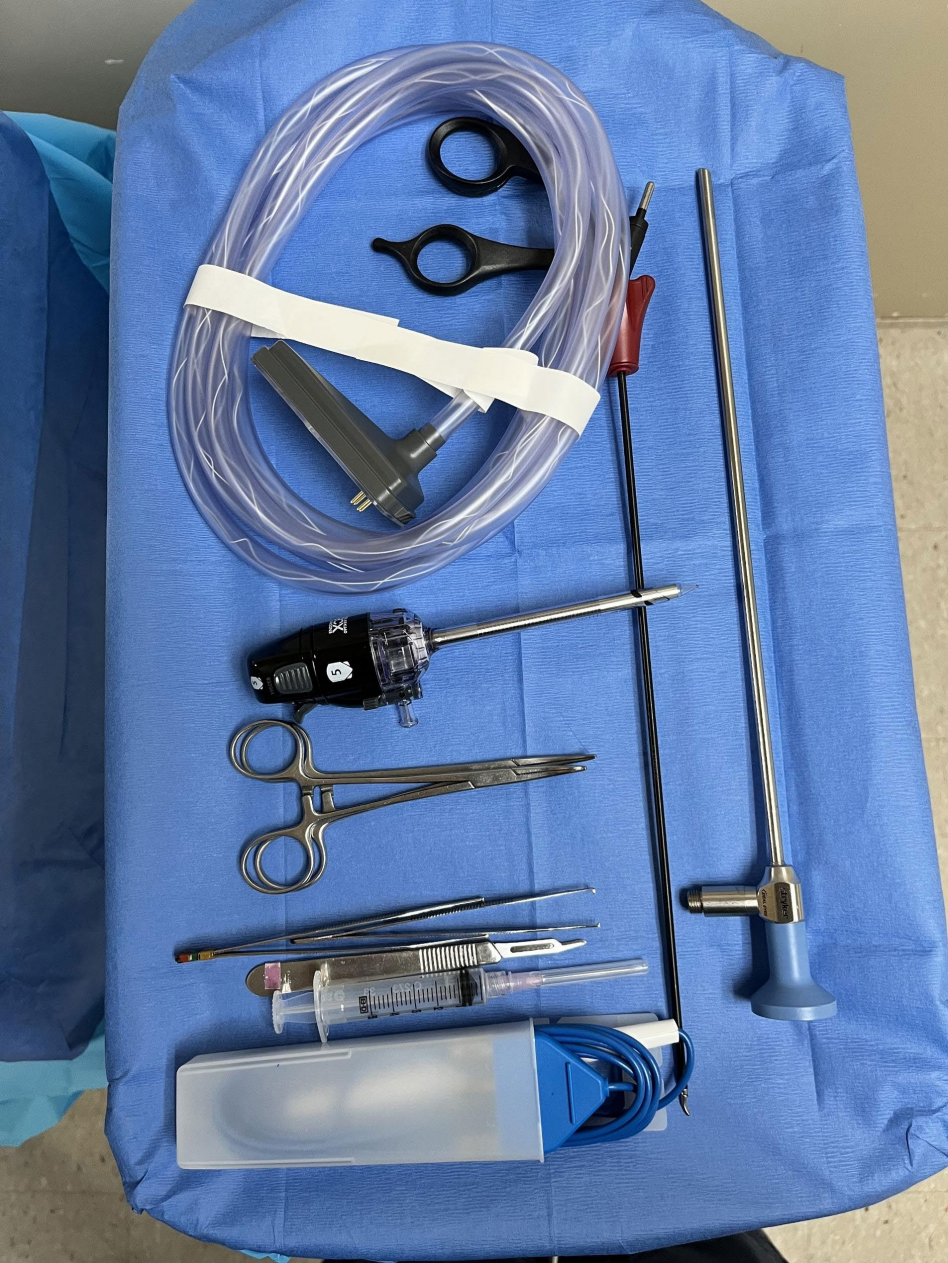
Laparoscopic equipment used for all hernia repairs. (**a**) Laparoscope 4.5 mm, 45-degree Hopkins lens. (**b**) Hi-Flow laparoscopic insufflator. (**c**) 5 mm trocar. (**d**) 3 mm Maryland grasper

All cases were performed under general anesthesia using inhaled anesthetics and intravenous medications, no preoperative medication was necessary, and no paralytics were administered. A Guatemalan-trained and certified pediatric anesthesiologist provided anesthesia for all cases. Laparoscopic equipment included a 4.5 mm, 45-degree Hopkins lens, a standard laparoscopic tower, an insufflator with a pediatric mode, and a 3 mm Maryland grasper (Fig. 1). Chloraprep was used for aseptic preparation of the skin. All repairs were performed via a modified extraperitoneal percutaneous closure (Patkowski-Takehara) technique. In the supine position, the abdomen was adequately prepped and draped. A small, vertical 5-mm incision was fashioned in the umbilicus through which a 5-mm port was placed, and the abdomen was insufflated to 15 mmHg pressure. A laparoscope was introduced into the peritoneal cavity. Visualization of the diagnosed inguinal hernia and examination of the contralateral side was done. A 3-mm incision was made on the contralateral abdominal wall through which a small Maryland grasper was introduced to aid with passing the needle pre-peritoneally. A 2-mm skin incision was made at the inguinal crease on the affected side without entering the abdominal cavity.

A single 3 − 0 prolene loop was deployed around the lateral aspect of the defect via a Touhy needle and used to pull a single 3 − 0 Ethibond suture circumferentially around the ring from the medial aspect of the defect. This approach is an amalgamation of the Patkowski percutaneous internal ring suturing, which relies on one port for visualization in the umbilicus, and the Takehara laparoscopic percutaneous peritoneal closure technique, in which a similar approach is taken but a grasping forceps is inserted in the contralateral abdominal wall [11, 12]. Our modification of this technique is the peritoneal cauterization that precedes the ring closure based on Novotny and colleagues’ “burnia.” This technique is meant to induce scarring and decrease hernia recurrence [13]. The ventral peritoneum was cauterized with a Maryland before tying down the Ethibond suture. The fascia was closed with running 2 − 0 Vicryl suture. All skin incisions were closed with a 5 − 0 fast-absorbing gut in a simple interrupted fashion. Local anesthetic (Bupivacaine) was injected into incision sites at the end of the case. All incisions were dressed with Dermabond. All patients received Ketorolac before awakening from anesthesia. Post-anesthesia recovery unit personnel administered oral acetaminophen or ibuprofen.

### Follow-up and data collection

Postoperatively, the primary investigator followed up with patients via telephone at the 1-week, 1-month, and 6-months. We decided to do a phone call-based follow-up because many families had traveled to the hospital from great distances limiting their ability to follow up in person after surgery.

The primary investigator contacted the patients and asked different sets of questions depending on the time since the operation. During the 1-week follow-up, families were asked how long it took for the patient to return to school if there were any signs or symptoms of infection, and to characterize postoperative pain. At the 1-month and 6-month follow-ups, families were queried on signs and symptoms of infection, postoperative pain, patient return to normal activities, and recurrence.

## Results

### Patient characteristics

A total of 14 patients underwent laparoscopic inguinal hernia repair during the study period. The mean age was 7.6 years (range 2–16). There was an equal distribution of gender; 7 participants were male and seven females. The right side was affected in 9 (64%) cases, while the left was in 4 (28%) cases. Only 1 (7%) case of bilateral hernias was documented. No patients were excluded from the study (Table 1).

**Table 1:** Patient characteristics

Age (years), mean	7.6
Male gender, n (%)	7 (50)
Side, n (%)	
Right	9 (64)
Left	4 (28)
Bilateral	1 (7)
Operative time (minutes), mean ± SD	28.07 ± 5.76
^a^LIHR male patient	27.33 ± 0.58
^b^RIHR male patient	28.67 ± 6.48
^c^BIHR male patient	NA^*^
LIHR female patient	24 (NA)
RIHR female patient	26.42 ± 5.8
BIHR female patient	28 (NA)

^a^Left inguinal hernia repair. ^b^ Right inguinal hernia repair. ^c^ Bilateral inguinal hernia repair. ^*^Not applicable

The mean operative time was 28.1 min (range 17–40), with no intraoperative complications. All patients were discharged within eight hours after surgery. Postoperative pain was managed with acetaminophen or ibuprofen; no patients required opioids.

At the 1-week follow-up, there were no cases of wound infection. Two patients (14%) reported mild postoperative pain that had to be managed with acetaminophen at home in the first three days after surgery; the rest of the cohort reported no use of NSAIDs or acetaminophen. Even though parents were instructed to send the patients to school the next day during postoperative education, the mean number of days off from school was two days.

At the 1-month follow-up, there were no infections, recurrence, or hydrocele formation cases. One patient reported difficulty returning to work as an agricultural worker. At 6-month follow-up, there were no reported infections or recurrences, and all patients returned entirely to normal activities. A summary of recorded outcomes is presented on Table 2.

**Table 2:** Recorded patient outcomes

Parameter	Number (%)
Operative complications	0 (0)
Surgical site infection	0 (0)
Post-operative pain	0 (0)
Recurrence at 6 months	0 (0)

## Discussion

Inguinal hernia is a common pediatric surgical problem worldwide; its repair is routine. Laparoscopic inguinal hernia repair is a minimally invasive approach that has become popular among surgeons due to its advantages. It includes comparable operative times, less postoperative pain, faster recovery times, and less recurrence rates to open repairs [1–3, 7]. The main advantage of laparoscopic repair is the ease of evaluation of the contralateral inguinal ring for a patent processus vaginalis allowing for repair and avoiding a second procedure in the case of a metachronous presentation of a contralateral hernia [6]. Our case series demonstrates that laparoscopic inguinal hernia repair is a safe and effective alternative to open inguinal hernia repair in LMICs. Despite the numerous benefits of laparoscopic inguinal hernia repair, the procedure’s use in Guatemala is limited. Our review of the literature leads us to believe this to be the first case series of its kind from Guatemala.

Minimally invasive surgery requires specialized equipment and trained personnel, often scarce in LMICs. Therefore, open repair is still the most common technique in surgical practice in these countries [10]. One of the significant challenges facing laparoscopic inguinal hernia repair is the cost of the procedure. The equipment required is expensive, and many small institutions in Guatemala cannot afford it. Another challenge that stands in the way of minimally invasive techniques in countries like Guatemala is the need for more infrastructure for the procedure. Many rural hospitals lack a proper ventilation system, have architectural limitations, or even lack reliable electricity to sustain power to the surgical devices. Despite these limitations, we performed this procedure in a small hospital, with only three operating rooms, connected to an orphanage in Patzun, Guatemala, with modern equipment.

Although the challenges mentioned before are barriers to adopting new surgical techniques, the most consequential component hindering surgical advancement in LMICs is the lack of human resources [10]. According to the Guatemalan Association of Pediatric Surgeons, approximately thirty surgeons with accredited training are currently practicing in the country. Guatemala has a population of children aged 0–14 of approximately 7 million, which means that general surgeons and not pediatric surgeons perform most pediatric surgery. Additionally, the learning curve for laparoscopic procedures can be steep, making adopting the technique much more difficult among providers; however, a 2013 study by Yoshizawa et al. analyzing the learning curve for the laparoscopic extraperitoneal hernia repair determined that attending surgeons needed a mean of twelve operations to perform the procedure safely and under 30 min and residents needed a mean of thirty procedures to achieve the same result [14]. These are encouraging findings for surgeons with little or no experience in pediatric laparoscopic surgery.

A 2023 survey by Tanoli et al. which included five Latin American countries, showed that there is a lack of investment from governments and hospitals in LMICs in laparoscopic surgery training and equipment because of perceived elevated healthcare costs even though much of the evidence suggests that healthcare costs are reduced longitudinally due to the lower morbidity exhibited by laparoscopy over open surgery. This same study determined that a shortage of basic supplies and insufficiently trained staff were the other top barriers to the adoption of laparoscopy [15].

**Fig. 2:**
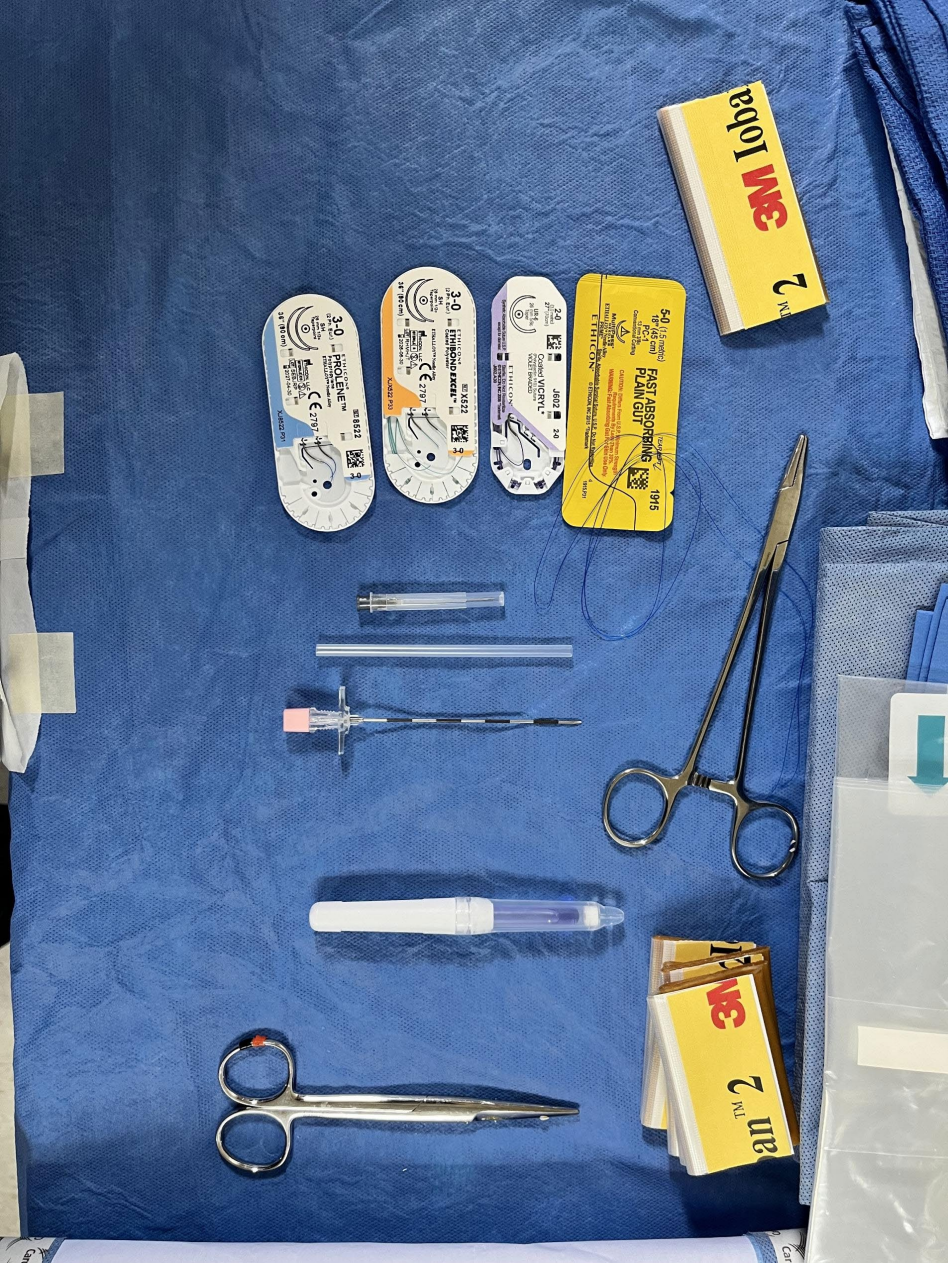
Surgical table setup. (**a**) Four suture packets used for the procedure. (**b**) Touhy needle. (**c**) Dermabond dressing

This medical mission involved carefully distributing six surgeons in three operating rooms and three procedure rooms. The operating room designated for pediatric surgery had an outdated anesthesia machine, a few power outlets, and medical air and oxygen outlets. A laparoscopic tower was donated to the International Esperanza Project and used for all cases. We faced the challenge of having limited resources forcing us to take a resource-saving approach. With only one Maryland grasper and two laparoscopes, we had to be strategic and make the most of what we had. To maximize efficiency, we decided only to open the necessary suture packets and individual instruments that were essential for the operation, thus allowing us to conserve resources and maintain a highly streamlined operation (Fig. 2). Despite these constraints, we delivered high-quality patient care, as evidenced by the series results. This experience demonstrates the feasibility of adopting new and state-of-the-art techniques considering resource management’s importance in a limited availability setting.

The advantages of laparoscopic inguinal hernia repair include less postoperative pain, faster recovery time, and a low rate of complications. The low rate of complications, including no infections or recurrence in the 6-month follow-up period, highlights the importance of proper surgical technique and postoperative care. The use of laparoscopy also allows for the identification of contralateral defects, which can be repaired simultaneously. The short operative time and same-day discharge indicate the feasibility of laparoscopic repairs in LMICs. However, access to surgeons knowledgeable in minimally invasive pediatric surgery and the equipment necessary to carry out the surgeries represent the most significant barrier to improving the standard of care in these countries.

The study had limitations. The sample is small, and it’s due to the team’s capacity to care for patients in the limited time we worked at the hospital. Since this was a mission trip, a larger sample could not be accrued beforehand, leading to an underpowered study. The decision to conduct patient follow-up through phone calls was made because most families were not locally based, and mobilization presented an important barrier to in-person follow-up. This study does not evaluate the effectiveness of laparoscopic inguinal hernia repair in the pediatric population; it merely states that it is possible to perform the procedure safely and effectively in a highly resource-limited setting and encourages the introduction of more advanced techniques in LMICs.

### Limitations

The study is not without limitations. The follow-up period was short and the sample size small. The reason behind is the amount of time we had in country to evaluate and treat these patients. As mentioned, in-person follow-up was very difficult because most families were not local and travelled great distances to get to the hospital. Aside from the economic burden of travel, transportation is complicated in rural Guatemala, with limited options.

## Conclusion

The results of this study were promising, with no intraoperative complications and short hospital stays. At the 6-month follow-up, there were no reported complications or recurrences, and all patients had returned to their normal activities. This study suggests that a laparoscopic approach to inguinal hernia repair can be safely and effectively used in LMICs, where access to laparoscopic surgery may be limited due to a lack of resources and surgical expertise. It is important to note that the study was conducted in a rural Guatemala institution with limited access to healthcare and surgical resources. Despite the challenges in this setting, the study demonstrates that laparoscopic inguinal hernia repair can be performed successfully in this context.

Overall, this study provides valuable insights into the use of laparoscopic inguinal hernia repair in LMICs and highlights the potential benefits of this technique for pediatric patients. Further longitudinal studies are needed to validate these early, positive outcomes and to assess the feasibility and cost-effectiveness of implementing laparoscopic inguinal hernia repair in other settings. Nonetheless, this technique provides a promising option for the surgical management of inguinal hernias in pediatric patients in Guatemala.

### Electronic supplementary material

Below is the link to the electronic supplementary material.

**Supplementary Table 1**: Study Participants

## Data Availability

All data supporting this study are available within the paper and its Supplementary Information.

## References

[1] Brandt ML (2008). Pediatric Hernias. Surg Clin North Am.

[2] Bowling K, Hart N, Cox P, Srinivas G, Online BMJ.). BMJ Publishing Group; 2017.

[3] Jessula S, Davies DA. Evidence supporting laparoscopic hernia repair in children. Current Opinion in Pediatrics. Volume 30. Lippincott Williams and Wilkins; 2018. pp. 405–10. 10.1097/MOP.0000000000000612 29461296

[4] Kantor N, Travis N, Wayne C, Nasr A (2019). Laparoscopic versus open inguinal hernia repair in children: which is the true gold-standard? A systematic review and meta-analysis. Pediatr Surg Int.

[5] Salma U, Aboel-Fetoh N, Mehmood Y, Alanizi ModhiA AO, AN OVERVIEW OF PEDIATRIC INGUINAL, HERNIAS: EXPERIENCE AT A TERTIARY CARE CENTER IN NORTHERN PROVINCE OF SAUDI ARABIA. Int J Adv Res (Indore) [Internet]. 2016;4(8):820–4. Available from: http://www.journalijar.com/article/10968/an-overview-of-pediatric-inguinal-hernias:-experience-at-a-tertiary-care-center-in-northern-province-of-saudi-arabia./.

[6] Suttiwongsing A, Khorana J, Ruangwongroj P, Niruttiwat K. Laparoscopic extraperitoneal technique versus open inguinal herniotomy in children: historical controlled intervention study. World J Pediatr Surg. 2022;5(4). 10.1136/wjps-2022-000436 PMC9716936 36474745

[7] Koivusalo AI. A review of the incidence, Manifestation, predisposing factors, and Management of Recurrent Pediatric Inguinal Hernia. European Journal of Pediatric Surgery. Volume 27. Georg Thieme Verlag; 2017. pp. 478–83. 10.1055/s-0037-1608675 29121686

[8] Choi W, Hall NJ, Garriboli M, Ron O, Curry JI, Cross K (2012). Outcomes following laparoscopic inguinal hernia repair in infants compared with older children. Pediatr Surg Int.

[9] Al-Taher RN, Khrais IA, Alma’aitah S, Al Saiad AA, Al-abboodi AA, Saleh OM et al. Is the open approach superior to the laparoscopic hernia repair in children? A retrospective comparative study. Annals of Medicine and Surgery. 2021;71. 10.1016/j.amsu.2021.102889 PMC8517708 34691442

[10] Krishnaswami S, Nwomeh BC, Ameh EA (2016). The pediatric Surgery workforce in low- and middle-income countries: problems and priorities. Semin Pediatr Surg.

[11] Patkowski D, Czernik J, Chrzan R, Jaworski W, Apoznański W. Percutaneous internal ring suturing: a simple minimally invasive technique for inguinal hernia repair in children. Journal of Laparoendoscopic & Advanced Surgical Techniques [Internet]. 2006;16(5):513–7. 10.1089/lap.2006.16.513. 10.1089/lap.2006.16.513 17004880

[12] Takehara H, Yakabe S, Kameoka K (2006). Laparoscopic percutaneous extraperitoneal closure for inguinal hernia in children: clinical outcome of 972 repairs done in 3 pediatric surgical institutions. J Pediatr Surg.

[13] Novotny NM, Puentes MC, Leopold R, Ortega M, Godoy-Lenz J (2017). The Burnia: laparoscopic sutureless inguinal hernia repair in girls. J Laparoendoscopic Adv Surg Techniques.

[14] Yoshizawa J, Ashizuka S, Kuwashima N, Kurobe M, Tanaka K, Ohashi S (2013). Laparoscopic percutaneous extraperitoneal closure for inguinal hernia: learning curve for attending surgeons and residents. Pediatr Surg Int.

[15] Tanoli O, Ahmad H, Khan H, Khan A, Aftab Z, Khan MI et al. Laparoscopy in Low- and Middle-Income Countries: A Survey Study. Cureus. 2023. 10.7759/cureus.40761 PMC10284685 37363112

