# Supplementary material for: Introduction of pediatric laparoscopic inguinal hernia repair in Guatemala
Source: BMC Surg. 2023 Nov 27;23:361. doi: 10.1186/s12893-023-02262-6 (PMC10683215; doi:10.1186/s12893-023-02262-6)
Supplement: Supplementary file 1 — Supplementary Table 1: Study Participants [file 12893_2023_2262_MOESM1_ESM.pdf]

Supplementary Table 1: Study participants

Patient Characteristics					Operative information	One week follow-up		30 day follow-up		6 month follow-up	
Date of Surgery	Gender	Age (years)	Weight (kgs)	Diagnosis	Operative time (mins)	Infection	Pain	Infection	Pain	Pain	Reocurrence
5/9/22	Male	16	55.9	[a]LIH	27	No infection	No pain	No	No	No	No
5/9/22	Male	2	10.5	[b]RIH	40	No infection	No pain	No	No	No	No
5/9/22	Male	7	33	LIH	27	No infection	No pain / playful	No	No	No	No
6/9/22	Female	5	14.5	RIH	20	No infection	No pain / playful	No	No	No	No
6/9/22	Male	13	42.3	RIH	27	No infection	No pain	No	No	No	No
6/9/22	Male	3	11.4	LIH	28	No infection	No pain / playful	No	No	No	No
6/9/22	Female	6	17.7	RIH	33	No infection	No pain	No	No	No	No
7/9/22	Male	13	40	RIH	27	No infection	No pain	No	No	No	No
7/9/22	Female	8	25	[c]BIH	28	No infection	No pain	No	No	No	No
7/9/22	Female	5	17	LIH	24	No infection	No pain	No	No	No	No
8/9/22	Female	10	26.4	RIH	30	No infection	No pain	No	No	No	No
8/9/22	Female	11	37.3	RIH	30	No infection	No pain	No	No	No	No
8/9/22	Female	5	19.5	RIH	17	No infection	No pain / itchy	No	No	No	No
8/9/22	Male	3	13.1	RIH	35	No infection	No pain / itchy	No	No	No	No

[a]Left inguinal hernia. [b] Right inguinal hernia. [c] Bilateral inguinal hernia.

The Supplementary Table 1 includes all the study participants and details some demographic information and clinical diagnosis. The follow-up columns include the two basic questions that came up every visit - infection and pain control.
